# Biplot evaluation of test environments and identification of mega-environment for sugarcane cultivars in China

**DOI:** 10.1038/srep15505

**Published:** 2015-10-22

**Authors:** Jun Luo, Yong-Bao Pan, Youxiong Que, Hua Zhang, Michael Paul Grisham, Liping Xu

**Affiliations:** 1 Key Laboratory of Sugarcane Biology and Genetic Breeding, Ministry of Agriculture, Fujian Agriculture and Forestry University, Fuzhou 350002, Fujian, China; 2 USDA-ARS, Sugarcane Research Unit, Houma, LA 70360, Louisiana, USA

## Abstract

Test environments and classification of regional ecological zones into mega environments are the two key components in regional testing of sugarcane cultivars. This study aims to provide the theoretical basis for test environment evaluation and ecological zone division for sugarcane cultivars. In the present study, sugarcane yield data from a three-year nationwide field trial involving 21 cultivars and 14 pilot test locations were analysed using both analysis of variance (ANOVA) and heritability adjusted-genotype main effect plus genotype-environment interaction (HA-GGE) biplot. The results showed that among the interactive factors, the GE interaction had the greatest impact, while the genotype and year interaction showed the lowest impact. Kaiyuan, Lincang and Baoshan of Yunnan, Zhangzhou and Fuzhou of Fujian, and Hechi, Liuzhou and Chongzuo of Guangxi, and Lingao of Hainan were ideal test environments with a demonstrated high efficiency in selecting new cultivars with a wide adaptability, whereas Baise of Guangxi was not. Based on HA-GGE biplot analysis, there are three ecological sugarcane production zones in China, the Southern China Inland Zone, the Southwestern Plateau Zone, and the Southern Coastal Zone. The HA-GGE biplot analysis here presents the ideal test environments and also identifies the mega-environment for sugarcane cultivars in China.

Environmental changes affect both crop growth and yield due to significant genotype × environment interactions (GE)^1^,^2^,^3^,^4^,^5^. The most reliable way to evaluate a cultivar is to grow it in multiple environments for several years^6^,^7^,^8^. The selection of suitable breeding and testing locations is crucial to the success of a plant breeding program. Besides, an ideal test location can not only be able to discriminate the genetic differences among genotypes, but also target environments for which selected genotypes are best adapted^9^,^10^. When evaluating the stability and adaptability of a cultivar, it is important to examine GE interaction and to assess its growth in different environments and ecological regions. Some cultivars are well adapted to specific ecological regions; that is, they show similarities in development potential and constraints under specific environments, or where the same group of cultivars forms the best combination year after year^11^,^12^,^13^,^14^,^15^,^16^. Due to its vegetative growth characteristics, the targeted production area of sugarcane is often divided into several relatively homogeneous ecological regions, which calls for a “regional breeding” scheme.

An additive main effect and multiplicative interaction (AMMI) model is commonly used to analyse GE interaction during yield trials. Understanding GE interaction is very important for evaluating the adaptability and stability of cultivars. AMMI can detect GE interaction in a multi-dimensional space and present the interaction using a biplot. AMMI has been used to analyse planting environments in wheat^17^,^18^, rice^19^, rapeseed^20^, and sugarcane^21^. However, AMMI biplot is not a true biplot and its application has been limited^22^,^23^. In contrast, the genotype main effect plus genotype-environment interaction (GGE) biplot model utilizes multi-region data for environmental evaluation and provides better graphical illustration^14^,^24^,^25^,^26^. GGE biplot can facilitate a better understanding of complex GE interaction in multi-environment trials of breeding lines and agronomic experiments. GGE biplot has been used to identify the performance of crop cultivars under multiple stress environments, ideal cultivars, mega-environment, and core testing sites^27^. It also has been successfully used in crop trials, including oats^28^, peanut^29^, rapeseed^30^, soybean^31^, wheat^32^, cotton^33^, sunflower^34^, and sugarcane^4^,^5^,^35^,^36^.

A HA-GGE biplot is a heritability adjusted-genotype main effect plus genotype-environment interaction biplot first reported by Yan and Holland in 2010^26^. In a HA-GGE biplot, an environmental discrimination power is approximately equal to the vector length of that environment, representativeness is approximately equal to the cosine of the angle between the environment vector and the average environment vector, and the desirability index is approximately equal to the projection of the environment vector onto the average environment vector axis^14^,^26^. A HA-GGE biplot can effectively analyse the GE interaction, identify the best cultivars for a specific ecological region, evaluate the test environments, and evaluate the desirability of a test environment based on its representativeness and discrimination power on genotypic differences^3^,^12^,^14^,^28^. Although there are more and more reports on the application of GGE biplot on sugarcane^4^,^5^,^35^,^36^, there is only one report on HA-GGE biplot application on sugarcane^37^.

In the present study, the HA-GGE biplot program was used to analyse the yield and GE interaction data from a three-year national sugarcane trial in China. The trial involved 21 cultivars and 14 environments across five provinces with more than 90% of the total sugarcane production areas in China. Discrimination power and representativeness of these test environments were also analysed to explore an ecological regionalization plan for these sugarcane cultivars. This study may provide the basis and support for selecting the best sugarcane cultivars to plant in a particular ecological region.

## Results

### Analysis of variance

Analysis of variance was performed for all the yield data from the same trial region or genotype during 2011 to 2013 (two plant-cane crops plus one ratoon crop). The results showed that the effects of different years, locations, genotypes, location × year, genotype × year, location × genotype, location × genotype × year were all highly significant (Table 1). Based on the percentage effect of each variant over the total effect (sum of squares), the relative contribution of various factors on yield variability were compared.

Environment (location) had the highest impact on yield, accounting for 40.40% of the yield variability (Table 1). The next was the GE interaction (genotype × location), accounting for 19.28%. The genotype alone accounted for the least variability (7.42%). Within the environment (location), changes in location, year, and location × year accounted for 29.79%, 0.43%, and 10.17% of the yield variance, respectively (Table 1). Overall, the impact of each factor on the yield variability could be ordered from high to low as: location (29.79%) > location × genotype (19.28%) > location × genotype × year (16.50%) > location × year (10.17%) > genotype (7.42%) > genotype × year (3.05%) > year (0.43%). Obviously, the last single factor (year) played a very minor role in the variability in yield.

### Discrimination power and representativeness of test environment (location)

**Figure 1:**
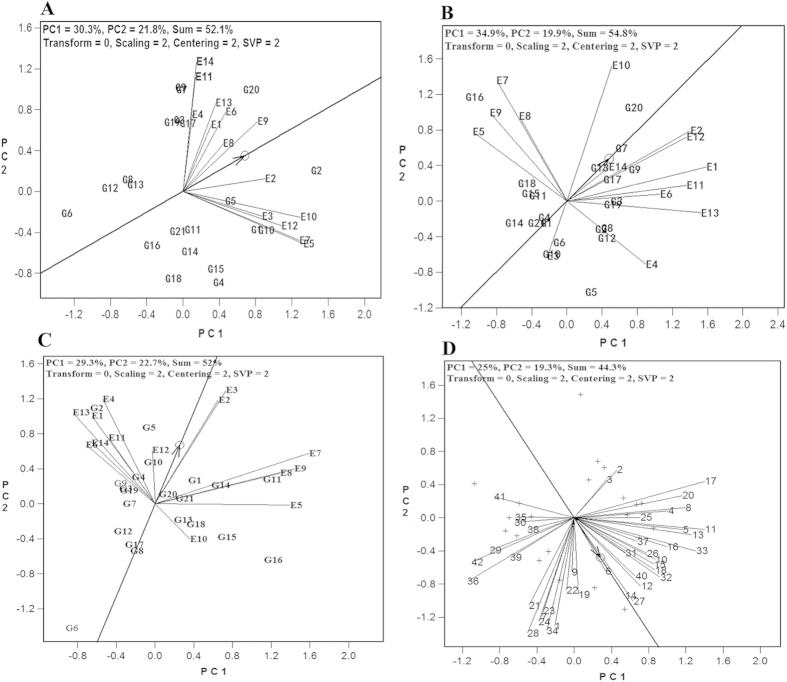
Correlation of yields with test environments based on HA-GGE biplot. (**A**) The first plant cane crop. (**B**) The second plant cane crop. (**C**), The first ratoon crop. (**D**), All crops. Numerical codes for cultivars: G1 = ROC22; G2 = DeZhe03–83; G3 = FuNong02–5707; G4 = FuNong0335; G5 = FuNong1110; G6 = FuNong36; G7 = FuNong39; G8 = GanNan02–70; G9 = LiuCheng03–1137; G10 = LiuCheng 05–136; G11 = MinTang01–77; G12 = YueGan34; G13 = YueGan35; G14 = YueGan40; G15  = YueGan42; G16 = YunRui 06–189; G17 = YunZhe04–241; G18 = YunZhe05–49; G19 = YunZhe05–51; G20 = YunZhe06–407; G21 = YunZhe06–80. Numerical codes for test environments: E1 = Fuzhou of Fujian; E2 = Zhangzhou of Fujian; E3 = Suixi of Guangdong; E4 = Zhanjiang of Guangdong; E5 = Baise of Guangxi; E6 = Chongzuo of Guangxi; E7 = Hechi of Guangxi; E8 = Laibing of Guangxi; E9 = Liuzhou of Guangxi; E10 = Lingao of Hainan; E11 = Baoshan of Yunnan; E12 = Kaiyuan of Yunnan; E13 = Lincang of Yunnan; E14 = Ruili of Yunnan. Numerical codes for year and location where 1yr, 2yr, and RA stand for first year planting, second year planting, and ratoon, respectively. 1, 2, and 3 = the first plant cane crop, the second plant cane crop, and the first ratoon crop at Lingao of Hainan (E10), respectively; 4, 5, and 6 = the first plant cane crop, the second plant cane crop, and the first ratoon crop at Baoshan of Yunnan, respectively; 7, 8, and 9 = the first plant cane crop, the second plant cane crop, and the first ratoon crop at Kaiyuan of Yunnan, respectively; 10, 11, and 12 = the first plant cane crop, the second plant cane crop, and the first ratoon crop at Lincang of Yunnan, respectively; 13, 14, and 15 = the first plant cane crop, the second plant cane crop, and the first ratoon crop at Ruili of Yunnan, respectively; 16, 17, and 18 = the first plant cane crop, the second plant cane crop, and the first ratoon crop at Fuzhou of Fujian, respectively; 19, 20, and 21 = the first plant cane crop, the second plant cane crop, and the first ratoon crop at Zhangzhou of Fujian, respectively; 22, 23, and 24 = the first plant cane crop, the second plant cane crop, and the first ratoon crop at Suixi of Guangdong, respectively; 25, 26, and 27 = the first plant cane crop, the second plant cane crop, and the first ratoon crop at Zhanjiang of Guangdong, respectively; 28, 29, and 30 = the first plant cane crop, the second plant cane crop, and the first ratoon crop at Baise of Guangxi, respectively; 31, 32, and 33 = the first plant cane crop, the second plant cane crop, and the first ratoon crop at Chongzuo of Guangxi, respectively; 34, 35, and 36 = the first plant cane crop, the second plant cane crop, and the first ratoon crop at Hechi of Guangxi, respectively; 37, 38, and 39 = the first plant cane crop, the second plant cane crop, and the first ratoon crop at Laibing of Guangxi, respectively; 40, 41, and 42 = the first plant cane crop, the second plant cane crop, and the first ratoon crop at Liuzhou of Guangxi, respectively.

The discrimination power of a test environment (location) in a HA-GGE biplot is proportional to the length of the environment vector, which is the line connecting the origin and the test environment point^14^,^26^. For the first plant cane crop, Baise of Guangxi (E5), Hechi of Guangxi (E7), and Lingao of Hainan (E10) showed relatively high values of discrimination power, while Fuzhou of Fujian (E1), Suixi of Guangdong (E3), Zhanjiang of Guangdong (E4), Chongzuo of Guangxi (E6), and Laibing of Guangxi (E8) showed relatively low values (Fig. 1A). For the second plant-cane crop, Hechi of Guangxi (E7), Lingao of Hainan (E10), Kaiyuan of Yunnan (E12), and Lincang of Yunnan (E13) showed relatively high values of discrimination power; Suixi of Guangdong (E3), Chongzuo of Guangxi (E6), and Laibing of Guangxi (E8) showed relatively low values (Fig. 1B). For the first ratoon crop, Suixi of Guangdong (E3), Baise of Guangxi (E5), Hechi of Guangxi (E7), and Liuzhou of Guangxi (E9) showed relatively high values of discrimination power, while Lingao of Hainan (E10), Baoshan of Yunnan (E11), and Kaiyuan of Yunnan (E12) showed relatively low values (Fig. 1C). When taking all three years’ data into consideration, Baise of Guangxi (E5), Hechi of Guangxi (E7), and Liuzhou of Guangxi (E9) showed relatively high values of discrimination power and Zhanjiang of Guangdong (E4), Chongzuo of Guangxi (E6), and Laibing of Guangxi (E8) showed relatively low values of discrimination power (Fig. 1D).

The representativeness of a test environment (location) refers to the consistency of a target environment when compared with other environments or the average of all test environments^9^,^10^. In a HA-GGE biplot, the representativeness of a target environment is shown by the angle between the test environment vector and the average environment coordination (AEC). AEC abscissa is a single-arrowed line (ray) passing through the biplot origin and the average of all environments^12^ (Fig. 1). The smaller the angle, the stronger the representativeness of the environment is^12^. For the first plant-cane crop, Zhangzhou of Fujian (E2), Chongzuo of Guangxi (E6), and Liuzhou of Guangxi (E9) showed relatively strong representativeness and Zhanjiang of Guangdong (E4), Baoshan of Yunnan (E11), and Ruili of Yunnan (E14) showed relatively weak representativeness (Fig. 1A). For the second plant-cane crop, Zhangzhou of Fujian (E2), Kaiyuan of Yunnan (E12), and Ruili of Yunnan (E14) showed relatively strong representativeness and Suixi of Guangdong (E3), Baise of Guangxi (E5), Hechi of Guangxi (E7), Laibing of Guangxi (E8), and Liuzhou of Guangxi (E9) showed relatively weak representativeness (Fig. 1B). For the first ratoon crop, Zhangzhou of Fujian (E2), Suixi of Guangdong (E3), Zhanjiang of Guangdong (E4), and Kaiyuan of Yunnan (E12) showed relatively strong representativeness and Baise of Guangxi (E5), Chongzuo of Guangxi (E6), Lingao of Hainan (E10), and Ruili of Yunnan (E14) showed relatively weak representativeness (Fig. 1C). Overall, Zhangzhou of Fujian (E2) showed a relatively strong representativeness, followed by Chongzuo of Guangxi (E6) and Kaiyuan of Yunnan (E12), whereas Baise of Guangxi (E5) showed a relatively weak representativeness (Fig. 1D).

### Test environment evaluation parameters

A HA-GGE biplot was used to analyse the parameters of the sugarcane trials including discrimination power, representativeness and the desirability index of the test environments. The data were standardized and evaluated comprehensively (Table 2). Based on the discrimination power of the test environment on the yield of various genotypes, the environments tested can be categorized as follows: Zhangzhou of Fujian (E2), Baise of Guangxi (E5), Hechi of Guangxi (E7), Liuzhou of Guangxi (E9), and Lincang of Yunnan (E13) had very strong discriminative test environments; Fuzhou of Fujian (E1), Lingao of Hainan (E10), Baoshan of Yunnan (E11), and Kaiyuan of Yunnan (E12) had strong discriminative test environments; Suixi of Guangdong (E3), Zhanjiang of Guangdong (E4), Chongzuo of Guangxi (E6), Laibing of Guangxi (E8), and Ruili of Yunnan (E14) had weak discriminative test environments.

Most test environments used in the trials showed strong representativeness, which include Fuzhou of Fujian (E1), Zhangzhou of Fujian (E2), Chongzuo of Guangxi (E6), Baoshan of Yunnan (E11), Kaiyuan of Yunnan (E12), Lincang of Yunnan (E13), and Ruili of Yunnan (E14). Overall the test environments showed good homogeneity, indicating that the trials were well-targeted and representative. Hechi, Laibing, and Liuzhou of Guangxi (E7, E8, and E9), Zhanjiang of Guangdong (E4), and Lingao of Hainan (E10) showed medium representativeness. Baise of Guangxi (E8) and Suixi of Guangdong (E3) showed weak representativeness. Test environments with medium to low representativeness may have special ecological condition(s) that require more careful and detailed trials for the selection of better cultivars.

The desirability index of a cultivar is a comprehensive evolution derived from the discrimination power and representativeness of the environment and thus is an important basis for selection of a test environment. Based on the discrimination power, test environments can be categorized as: (1) the ideal environments, e.g. Zhangzhou of Fujian (E2), Fuzhou of Fujian (E1), Chongzuo of Guangxi (E6), Hechi of Guangxi (E7), Liuzhou of Guangxi (E9), Lingao of Hainan (E10), Baoshan of Yunnan (E11), Kaiyuan of Yunnan (E12), and Lincang of Yunnan (E13); (2) relatively ideal environments, e.g. Suixi of Guangdong (E3), Zhanjiang of Guangdong (E4), Laibing of Guangxi (E8), and Ruili of Yunnan (E14); and (3) undesirable environments, e.g. Baise of Guangxi (E5).

### The best test environment (location) for sugarcane cultivars

**Figure 2:**
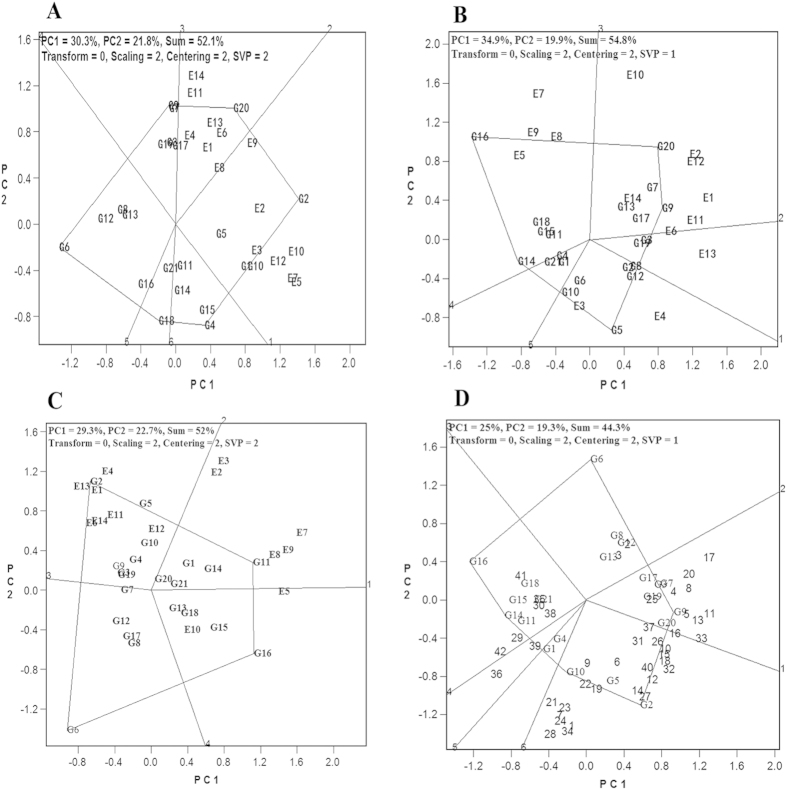
HA-GGE biplot of cane yields from sugarcane national regional trials. (**A**) The first plant cane crop. (**B**) The second plant cane crop. (**C**), The first ratoon crop. (**D**), All crops. Numerical codes are provided in Fig. 1 legend.

The 14 test environments could be divided into two groups based on the “which-won-where” pattern of the HA-GGE biplot from the first plant-cane trials (Fig. 2A). The best performer was YZ06–407 (G20) at Fuzhou of Fujian (E1), Zhanjiang of Guangdong (E4), Chongzuo of Guangxi (E6), Baoshan of Guangxi (E11), Lincang of Yunnan (E13), and Ruili of Yunnan locations (E14). The worst performers were DZ03–83 (G2) at Zhangzhou of Fujian (E2), Suixi of Guangdong (E3), Baise of Guangxi (E5), Hechi of Guangxi (E7), Laibing of Guangxi (E8), Liuzhou of Guangxi (E9), Lingao of Hainan (E10), and Kaiyuan of Yunnan (E12) locations. For the second plant-cane trials (Fig. 2B), there were three best performing cultivars. YZ06–407 (G20) performed the best at Fuzhou of Fujian (E1), Zhangzhou of Fujian (E2), Chongzuo of Guangxi (E6), Lingao of Hainan (E10), Baoshan of Yunnan (E11), Kaiyuan of Yunnan (E12), Lincang of Yunnan (E13), and Ruili of Yunnan (E14) locations. YR06–189 (G16) performed the best in Baise of Guangxi (E5), Hechi of Guangxi (E7), Laibing of Guangxi (E8) and Liuzhou of Guangxi (E9). FN1110 (G5) performed the best in Suixi of Guangdong (E3) and Zhanjiang of Guangdong (E4). For the first ratoon crop trials (Fig. 2C), the 14 test environments (locations) were divided by two best performing cultivars. DZ03–83 (G2) performed the best in Fuzhou of Fujian (E1), Zhanjiang of Guangdong (E4), Chongzuo of Guangxi (E6), Baoshan of Yunnan (E11), Kaiyuan of Yunnan (E12), Lincang of Yunnan (E13), and Ruili of Yunnan (E14). MT01–77 (G11) performed the best in Zhangzhou of Fujian (E2), Suixi of Guangdong (E3), Baise of Guangxi (E5), Hechi of Guangxi (E7), Labing of Guangxi (E8), and Liuzhou of Guangxi (E9). Based on all data from the three year trials, DZ03–83 (G2), FN1110 (G5), LC03–1137 (G9), LC05–136 (G10), and YZ06–407 were good performers at most of the test locations (Fig. 2D).

### Ecological regionalization of sugarcane cultivars based on HA-GGE biplot

Ecological regionalization of cultivars requires the construction and analysis of a HA-GGE biplot from multiple test locations. When several reproducible test locations are identified, an ecological regionalization plan of the cultivars can be summarized^12^,^14^,^16^. Since GE interaction can be affected by many factors, it is difficult to obtain identical test locations and ecological regionalization of cultivars, which may have to be deduced from multiple sets of test data from the same location. In this regard, several combined locations or in other words, ecological regions (zones) were explored. Firstly, the reproducibility of a test group was inferred from the probability of the corresponding locations being placed within the same group^16^. Secondly, a HA-GGE biplot was used to analyse trial data from locations of similar groups. Then the best cultivars and locations in the corresponding sectors were determined. Based on the results of the 14 test locations, Baise of Guangxi (E5), Hechi of Guangxi (E7), Laibing of Guangxi (E8), and Liuzhou of Guangxi (E9) were considered to belong to the same ecological region representing the Southern China Inland Sugarcane Production Zone; Baoshan of Yunnan (E11), Kaiyuan of Yunnan (E12), Lincang of Yunnan (E13), and Ruili of Yunnan (E14) to belong to the same ecological region representing the Southwestern Plateau Sugarcane Production Zone; and Fuzhou of Fujian (E1), Zhanjiang of Guangdong (E4), Chongzuo of Guangxi (E6), Zhangzhou of Fujian (E2), Suixi of Guangdong (E3), and Lingao of Hainan (E10) to belong to the same ecological region representing the Southern China Coastal Production Zone.

### High-yielding and yield stability of the cultivars

**Figure 3:**
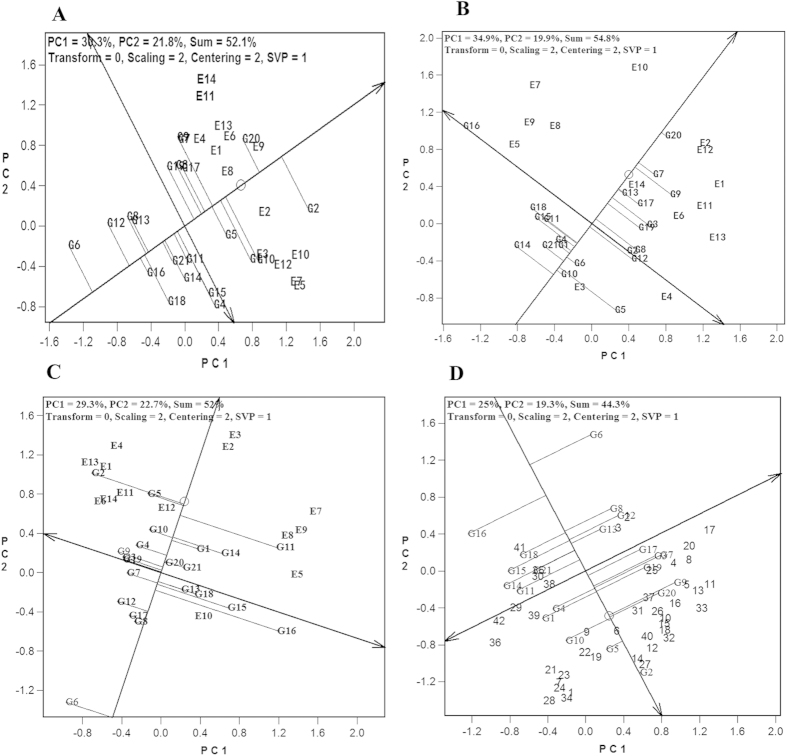
GGE-based yield and stability analysis of 21 sugarcane cultivars. (**A**) The first plant cane crop. (**B**) The second plant cane crop. (**C**) The first ratoon crop. (**D**) All crops. Numerical codes are provided in Fig. 1 legend.

In Fig. 3A, the first and second principal components (PC1 and PC2) of the yield traits accounted for G (30.3%) and GE (21.8%), or GGE (52.1%) combined, for the first plant-cane trials. DZ03–83 (G2) had the highest average cane yield, followed by YZ06–407 (G20) and LC05–136 (G10). The check ROC22 (G1) ranked the fourth, followed by LC03–1137 (G9), FN39 (G7), FN1110 (G5), YZ04–241 (G17), FN02–5707 (G3), and YZ05–51 (G19). These six cultivars produced greater than the average cane yields but less than the check. When the yield stability was taken into account, YZ06–407 (G20) and FN1110 (G5) both had high yields and high stability over all the other cultivars.

In Fig. 3B, the PC1 and PC2 of the yield traits accounted for G (34.9%) and GE (19.9%), or GGE (54.8%) combined, for the second plant-cane trials. YZ06–407 (G20) produced the highest average cane yield, followed by FN39 (G7) and LC03–1137 (G9). Other cultivars that produced higher than average yield and the yield of the check ROC22 (G1) included YZ04–241 (G17), YG35 (G13), FN02–5707 (G3), YZ05–51 (G19), GN02–70 (G8), and DZ03–83 (G2). When the yield stability was also taken into account, YZ06–407 (G20), FN39 (G7), YG35 (G13), and YZ04–241 (G17) were ideal cultivars with high yield and high stability over all the other cultivars.

In Fig. 3C, the PC1 and PC2 of the yield traits accounted for G (29.3%) and GE (22.7%), or GGE (52.0%) combined, for the first ratoon crop trials. DZ03–83 (G2) produced the highest average cane yield, followed by FN1110 (G5), MT01–77 (G11), YG40 (G14), and LC05–136 (G10). The check ROC22 (G1) ranked the sixth. FN0335 (G4), YZ06–407 (G20), YZ06–80 (G21), LC03–1137 (G9), FN02–5707 (G3), and YZ05–51 (G19) produced yields that were more than the average, yet less than the yield of ROC22 (G1). When the yield stability was also taken into account, FN1110 (G5) and LC05–136 (G10) produced stable higher yields over all the other cultivars in the first ratoon crop. When combining the yield data of all three years together, DZ03–83 (G2), FN1110 (G5), LC05–136 (G10), YZ06–407 (G20), and LC03–1137 (G9) yielded better than the Check ROC22 (G1). YZ05–51 (G19), FN39 (G7), FN0335 (G4), and FN02–5707 (G3) produced higher than the average, but lower than the check, yields. Again, DZ03–83 (G2) and FN1110 (G5) produced stable higher yields over all the other cultivars including ROC22 (G1) (Fig. 3D).

## Discussion

The GGE biplot is being used globally. It provides an effective statistical analysis approach for analysing the effects of GE interaction in crop regional trials^23^,^24^,^25^. As an upgraded version of GGE, HA-GGE biplot is being used for evaluating and screening of test environments^12^. Its graphic parameters are directly associated with the parameters of traditional quantitative genetics, which makes the analysis of relationship and interaction feasible between different parameters^13^,^14^,^15^,^16^. A HA-GGE biplot intuitively displays information regarding the yield and yield stability of each cultivar as well as other parameters such as discrimination power, representativeness, and desirability of the targeted environments. It also shows numerical results based on these parameters^12^,^14^,^38^. Test environments are dynamic factors that fluctuate considerably between years^39^,^40^,^41^. Therefore, when using a HA-GGE biplot to analyse GE interaction and define ecological regions for planting cultivars, it is necessary to perform analysis based on test data from multiple years and regions.

Ramburan *et al.* (2012) integrated empirical and analytical approaches to investigate sugarcane genotype × environment interaction by using variance components, GGE biplot, and AMMI. They found that environmental covariates and genotypic traits were correlated to AMMI scores and superimposed on biplots^35^. Besides, the G × E interaction accounted for more variation than the main effect of genotype^35^. Glaz and Kang (2008) investigated the location contributions via GGE biplot analysis of multi environment sugarcane genotype-performance trials^36^. Through assessing the contributions of a sand-soil location to the final stage of multi-environment testing of sugarcane genotypes in Florida, they concluded that it is desirable to replace an organic-soil location with a sand-soil location in the final testing stage of this sugarcane breeding and selection program^36^. In the present study, HA-GGE biplot analysis showed that the test environments had a greater effect on cane yield than either genotype or GE interaction alone. Among the interactive parameters, the Location × Genotype interaction had the greatest effect, whereas the Genotype × Year had the least effect. The extent of effect on cane yields was Location (29.79%) > Location × Genotype (19.28%) > Genotype × Location × Year (16.50%) > Location × Year (10.17%) > Genotype (7.42%) > Genotype × Year (3.05%) > Year (0.43%). The GE interaction effect was far greater than the genotype effect alone and some sugarcane cultivars may only adapt to certain specific locations. Therefore, sugarcane breeders are advised to increase the number of cultivars in evaluation tests, whenever possible, so long as the local ecological conditions allow. In addition, the regional layout of these cultivars should be such that the best fit cultivars based on rational regional distribution are planted in the most desirable environments to maximize positive GE interaction effects.

Another aim of regional variety tests is to identify ecological zones by evaluating the test environments^14^. The GE interaction effect needs to be considered when recommending ecological regions for planting certain cultivars^19^,^42^. The significance of a cultivar evaluation may be decreased if based on either its average performance across the entire ecological zones alone or its performance in nearby test regions alone^38^. This problem can be circumvented by using the HA-GGE biplot program to visually display yield, yield stability, and discriminative power of a test environment. The ability to identify test regions with good discrimination power will help improve the accuracy and efficiency of regional trials^12^,^14^. If all cultivars produce low yields without any significant difference within a test region, it is mostly caused by factors related to human management or natural disasters. An important usefulness of GGE biplot is to identify redundant testing locations and if the redundant locations are removed, precision and important information about the cultivars will not be sacrificed^41^. Therefore, to evaluate the representativeness and discrimination power of a test region, it is necessary to perform long-term tests and analyse the data collected from year to year to minimize factors related to human management or natural disasters.

According to previous reports, a desirable region for a cultivar can be identified by comparing the discrimination power and representativeness of all the regions tested^40^,^41^. Luo *et al.*^4^,^5^ used GGE biplot to analyse data from sugarcane trials involving seven cultivars tested under seven environments. They identified Chongzuo of Guangxi and Lincang of Yunnan as two unique regions for further yield trials. Here we report yield data from a three-year trial involving two years of plant-cane crops and one year of ratoon crop. Out of 14 test locations, Zhangzhou of Fujian (E2), Fuzhou of Fujian (E1), Chongzuo of Guangxi (E6), Hechi of Guangxi (E7), Liuzhou of Guangxi (E9), Lingao of Hainan (E10), Baoshan of Yunnan (E11), Kaiyuan of Yunnan (E12), and Lincang of Yunnan (E13) were found to be the ideal test environments for sugarcane cultivar evaluations. These are ideal regions with a demonstrated high efficiency in selecting new cultivars with a wide adaptability. Suixi of Guangdong (E3), Zhanjiang of Guangdong (E4), Laibing of Guangxi (E8), and Ruili of Yunnan (E14) were relatively less ideal environments, while Baise of Guangxi (E5) was found to be an undesirable environment for cultivar selection with a wider adaptability. In general, cultivars selected from ideal environments are most likely the ones with outstanding average performance in all or most of the test regions with a wider adaptability.

Yan *et al.* (2011) used GGE biplot to analyse the mega-environments and test-locations for oat in Quebec^42^. They revealed that the Quebec oat-growing regions can be successfully divided into two distinct mega-environments^42^. At the conclusion of the study, we were able to divide the Chinese sugarcane production regions into three major ecological zones represented by the 14 test locations: 1) the Southern China Inland Sugarcane Production Zone represented by Baise (E5), Hechi (E7), Laibing (E8), and Liuzhou (E9) of Guangxi; 2) the Southwestern Plateau Sugarcane Production Zone represented by Baoshan (E11), Kaiyuan (E12), Lincang (E13), and Ruili (E14) of Yunnan; and 3) the Southern China Coastal Sugarcane Production Zone represented by Fuzhou (E1) and Zhangzhou (E2) of Fujian, Suixi (E3) and Zhanjiang (E4) of Guangdong, Chongzuo (E6) of Guangxi, and Lingao (E10) of Hainan. This finding is similar to our previous report^37^. Currently in China, the evaluation of new sugarcane cultivars is based on the average performance of the cultivars in their target regions, using a “one-type-fits-all” screening method^4^,^5^. Using this strategy, the breeders had to take all sugarcane growing areas as the target environments. Sugarcane cultivars selected from one ecological zone often do not perform well in the other ecological zone. As a result of this targeted regional evaluation and selection, each ecological zone might not have the most suitable cultivars to plant. Moreover, the “one-type-fits-all” cultivars may pose potential risks even when they are grown in the most suitable regions^37^,^43^. Therefore, an appropriate adjustment on test environments and evaluation criteria is always necessary to define ecological zones more accurately and to further improve the effectiveness of variety trials^40^,^41^. For example, the Southwestern Plateau Sugarcane Production Zone is located in very different geological areas under very different climates from the other two sugarcane production zones. In order to promote sugarcane production in the Southwestern China Plateau Sugarcane Production Zone, breeders should focus on selecting and promoting cultivars that are well adapted to that ecological zone. Unfortunately, currently most of the Chinese sugarcane breeding programs are located in the Southern Coastal Sugarcane Production Zone. Therefore, if one hopes to breed the cultivars suitable to this zone, then more sugarcane cultivars need to be test, other than the limited number of cultivars entering the national regional trail. Previous research also demonstrated that due to the large effect of genotype by mega-environment interaction, cultivar evaluation must be conducted specifically to each mega-environment prior to cultivar recommendation^42^. To address this issue, sugarcane breeding activities need to be intensified in other two ecological zones, namely, the Southern China Inland Sugarcane Production Zone and the Southwestern China Plateau Sugarcane Production Zone.

In conclusion, among the interactive factors, Location (region) × Genotype interaction showed the greatest effect and Genotype × Year showed the least impact on sugarcane yields. Based on the HA-GGE biplots, Zhangzhou of Fujian (E2), Fuzhou of Fujian (E1), Chongzuo of Guangxi (E6), Hechi of Guangxi (E7), Liuzhou of Guangxi (E9), Lingao of Hainan (E10), Baoshan of Yunnan (E11), Kaiyuan of Yunnan (E12), and Lincang of Yunnan (E13) were the ideal test environments for the selection of widely adaptable high yielding sugarcane cultivars, whereas Baise of Guangxi (E5) was an undesirable environment. Besides, Suixi of Guangdong (E3), Zhanjiang of Guangdong (E4), Laibing of Guangxi (E8), and Ruili of Yunnan (E14) were relatively less ideal test environments. This study was also able to divide the Chinese sugarcane production regions into three major ecological zones represented by the 14 test locations, the Southern China Inland Ecological Cultivation Zone, the Southwestern Plateau Ecological Cultivation Zone and the Southern China Coastal Ecological Cultivation Zone. Based on the overall results, two cultivars, DZ 03–83 (G2) and FN 1110 (G5), produced stable higher yields than the other 19 cultivars including the check ROC 22 (G1). The yield of DZ 03–83 (G2), FN 1110 (G5), LC 05–136 (G10), YZ 06–407 (G20) and LC03–1137 (G9) was higher than the check. The yield of YZ 05–51 (G19), FN 39 (G7), FN 0335 (G4) and FN 02–5707 (G3) was higher than average but lower than the check. DZ 03–83 (G2) and FN 1110 (G5) are ideal cultivars with high yield and great stability.

## Methods

### Ethics Statement

We confirm that no specific permits were required for the described locations/activities. We also confirm that the field studies did not involve any endangered or protected species.

### Ecological regions and sugarcane cultivars tested

Fourteen test environments (locations) were selected in the five provinces in China, namely, Fujian (FJ), Guangdong (GD), Guangxi (GX), Hainan (HN), and Yunnan (YN), and accounting for more than 90% of the total sugarcane production areas in China. These locations were Fuzhou of Fujian (FZFJ, E1), Zhangzhou of Fujian (FJZZ, E2), Suixi of Guangdong (GDSX, E3), Zhanjiang of Guangdong (GDZJ, E4), Baise of Guangxi (GXBS, E5), Chongzuo of Guangxi (GXCZ, E6), Hechi of Guangxi (GXHC, E7), Laibing of Guangxi (GXLB, E8), Liuzhou of Guangxi (GXLZ, E9), Lingao of Hainan (HNLG, E10), Baoshan of Yunnan (YNBS, E11), Kaiyuan of Yunnan (YNKY, E12), Lincang of Yunnan (YNLC, E13), and Ruili of Yunnan (YNRL, E14). Table 3 shows the longitude, latitude, altitude, soil type, precipitation and environmental parameters of these test ecological locations^37^. Twenty-one sugarcane cultivars were evaluated, including ROC 22, the check (G1), DZ 03–83 (G2), FN 02–5707 (G3), FN 0335 (G4), FN 1110 (G5), FN 36 (G6), FN 39 (G7), GN 02–70 (G8), LC 03–1137 (G9), LC 05–136 (G10), MT 01–77 (G11), YG 34 (G12), YG 35 (G13), YG 40 (G14), YG 42 (G15), YR 06–189 (G16), YZ 04–241 (G17), YZ 05–49 (G18), YZ 05–51 (G19), YZ 06–407 (G20) and YZ 06–80 (G21). ROC 22, a prevailing sugarcane cultivar in China, was included as a check.

### Trial design

Cultivar trials were conducted during 2011 to 2013 using a randomized complete block design with three replications. Each block had four 8 m-long rows with a 1.1 m space between rows, covering an area of 35.2 m^2^. During 2011 to 2012, the first plant-cane crop was evaluated; during 2012 to 2013, the second plant-cane crop was evaluated; and during 2012 to 2013, the first ratoon crop from the two plant-cane crops was evaluated. Planting was conducted during late February to early March in each year with a rate of 105,000 stalks per ha. Field management was slightly better than adjacent commercial fields, including timely intertill hilling, fertilization, irrigation, and pest control. The fertilizers applied had a N:P:K ratio of 4.6:1.6:1.0, with nitrogen fertilizer at 345 kg/ha, phosphorus fertilizer at 240 kg/ha, and potassium fertilizer at 78 kg/ha. A base fertilizer that accounted for 40% of the total was applied at the beginning of planting season. During elongation stage in early/middle/or late of July, a top dressing fertilizer that accounted for 60% of the total was applied in conjunction with intertill hilling. Every field management practice was performed on the same day for each test region. Yield was measured prior to final harvest. The plants of the middle two rows of each block was harvested and weighed, the area of the harvest was measured, and the number of stalks was counted. The cane yield of each block was then calculated based on the harvest area, the number of stalks harvested and the total weight of stalks from the harvested rows.

### Data processing

DPS v14.10 statistical analysis software was used for analysis of variance (ANOVA)^44^,^45^. The GGE-Biplot software was used for HA-GGE biplot analysis^14^,^26^. Yield trait data from multiple plot sites were summarized into a sugarcane cultivar-plot site two-way table, in which each value is the average trait value of the corresponding sugarcane cultivar at the corresponding trial site. The general model for GGE biplot is^26^:


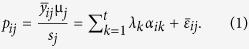


The response (*G*) observed in target environment j’ due to indirect selection in test environment j is^26^:


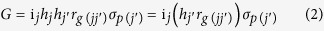


From Eq. 1, the usefulness of the test environment in indirect selection for the target environment has to be evaluated with regard to two aspects: (1) the heritability for the trait of interest in the environment (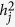), and (2) its genetic correlation with the target environment (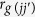).

A HA-GGE biplot judges correlation using the cosine of the angle between two vectors^14^,^26^. The projection of a sugarcane cultivar or trial environment vector on the AT axis (average-tester axis, which is the average environment vector) is used to judge the average performance of the cultivar or the desirability of the environment. The distance of the sugarcane cultivar or trial environment vector to the AT axis is used to judge the stability of the cultivar or the representativeness of the environment^14^.

In the HA-GGE biplot of a “suitable combination of genotype and environment” functional diagram or the “which-won-where” pattern, the peripheral cultivars were connected in turn to form a polygon^14^,^26^. All other cultivars were in the polygon. The perpendicular lines from the origin of the biplot to the sides of the polygon divide the polygon into fan-shaped sectors^14^,^26^. The cultivars in the same sector constitute a test combination. Within each sector, the cultivar located at the polygon vertex is the one with best average performance, which means it is the best cultivar in the test group^14^,^26^.

## Additional Information

**How to cite this article**: Luo, J. *et al.* Biplot evaluation of test environments and identification of mega-environment for sugarcane cultivars in China. *Sci. Rep.* **5**, 15505; doi: 10.1038/srep15505 (2015).

## Figures and Tables

**Table 1:** Combined analysis of variance (ANOVA) of sugarcane yields.

Source of variation	df	SS	MS	F	Percentage of treatment SS/%
E	41	542500.70	13231.73	140.43^**^	40.40
G	20	99638.57	4981.93	52.87^**^	7.42
G × E	820	521492.04	635.97	6.74^**^	38.83
R /E	84	20987.74	249.85	2.65^**^	1.56
(G×R)/E	1680	158295.61	94.22		
TOTAL	2645	1342914.92			
L	13	400117.42	30778.26	5.89^**^	29.79
Y	2	5777.21	2888.60	28.79^**^	0.43
Y×L	26	136606.32	5254.09	55.76^**^	10.17
R/E	84	20987.75	249.85		1.56
G	20	99638.57	4981.93	4.87^**^	7.42
G×L	260	258957.45	995.99	2.34^**^	19.28
G×Y	40	40898.00	1022.45	10.85^**^	3.05
Y×G×L	520	221636.59	426.22	4.52^**^	16.50
(G×R)/E	1680	158295.61	94.22		
Total	2645	1342914.92			

*P<0.05; **P<0.01.

**Table 2:** Standardized test location evaluation parameters.

Test Location	Discriminating power (mean ± *SD*)	Representativeness (mean ±*SD*)	Desirability index (mean ± *SD*)
E1	1.19 ± 0.46	0.75 ± 0.14	0.89 ± 0.44
E2	1.29 ± 0.35	0.97 ± 0.02	1.25 ± 0.35
E3	1.02 ± 0.43	0.27 ± 1.03	0.52 ± 1.04
E4	1.07 ± 0.28	0.47 ± 0.31	0.50 ± 0.40
E5	1.38 ± 0.05	0.28 ± 0.42	0.41 ± 0.58
E6	0.99 ± 0.08	0.66 ± 0.25	0.65 ± 0.24
E7	1.55 ± 0.16	0.52 ± 0.24	0.81 ± 0.37
E8	1.03 ± 0.35	0.60 ± 0.34	0.56 ± 0.25
E9	1.30 ± 0.21	0.54 ± 0.46	0.67 ± 0.52
E10	1.16 ± 0.55	0.41 ± 0.75	0.74 ± 0.89
E11	1.13 ± 0.24	0.65 ± 0.13	0.76 ± 0.30
E12	1.10 ± 0.47	0.86 ± 0.13	0.95 ± 0.47
E13	1.27 ± 0.31	0.64 ± 0.13	0.79 ± 0.17
E14	0.95 ± 0.33	0.66 ± 0.29	0.58 ± 0.15

**Table 3:** Ecological information of the 14 test locations.

Location	Longitude (E)	Latitude (N)	Altitude (m)	Soil type	Precipitation (mm)	Annual daylenth (h)	Mean daily temperature ^o^C)
E1	119.38	26.08	10.00	Sandy soil	**1600**	**1700**	**18.6**
E2	117.35	24.52	12.84	Sandy soil	1500	2000	21.0
E3	110.25	21.23	50.00	Sandy loam	1759	1864	24.2
E4	110.26	21.16	22.00	Red loam	1691	2106	23.0
E5	106.98	23.68	82.50	Sandy soil	1100	1448	21.0
E6	108.55	22.94	78.00	Loam soil	1200	1600	20.8
E7	108.06	24.73	110.00	Red loam	1500	1696	20.2
E8	109.08	23.76	95.00	Sandy soil	1400	1750	20.8
E9	109.36	24.47	99.10	Yellow soil	1700	1570	20.0
E10	109.69	19.92	20.00	Red loam	1417	2349	24.5
E11	99.01	25.02	670.00	Sandy soil	1000	2307	21.3
E12	103.25	23.70	1055.00	Sandy soil	**700**	**2200**	**19.8**
E13	99.95	24.15	1030.00	Red loam	1200	2257	19.6
E14	97.85	24.01	780.00	Loam soil	1355	2330	21.2
